# Tumor Segmentation in Contrast-Enhanced Magnetic Resonance Imaging for Nasopharyngeal Carcinoma: Deep Learning with Convolutional Neural Network

**DOI:** 10.1155/2018/9128527

**Published:** 2018-10-17

**Authors:** Qiaoliang Li, Yuzhen Xu, Zhewei Chen, Dexiang Liu, Shi-Ting Feng, Martin Law, Yufeng Ye, Bingsheng Huang

**Affiliations:** ^1^School of Biomedical Engineering, Health Science Centre, Shenzhen University, Shenzhen, China; ^2^Department of Radiology, Guangzhou Panyu Central Hospital, Guangzhou, China; ^3^Medical Imaging Institute of Panyu, Guangzhou, China; ^4^Department of Radiology, First Affiliated Hospital, Sun Yat-Sen University, Guangzhou, China; ^5^Department of Radiology, Queen Mary Hospital, Hong Kong

## Abstract

**Objectives:**

To evaluate the application of a deep learning architecture, based on the convolutional neural network (CNN) technique, to perform automatic tumor segmentation of magnetic resonance imaging (MRI) for nasopharyngeal carcinoma (NPC).

**Materials and Methods:**

In this prospective study, 87 MRI containing tumor regions were acquired from newly diagnosed NPC patients. These 87 MRI were augmented to >60,000 images. The proposed CNN network is composed of two phases: feature representation and scores map reconstruction. We designed a stepwise scheme to train our CNN network. To evaluate the performance of our method, we used case-by-case leave-one-out cross-validation (LOOCV). The ground truth of tumor contouring was acquired by the consensus of two experienced radiologists.

**Results:**

The mean values of dice similarity coefficient, percent match, and their corresponding ratio with our method were 0.89±0.05, 0.90±0.04, and 0.84±0.06, respectively, all of which were better than reported values in the similar studies.

**Conclusions:**

We successfully established a segmentation method for NPC based on deep learning in contrast-enhanced magnetic resonance imaging. Further clinical trials with dedicated algorithms are warranted.

## 1. Introduction

Head and neck cancer (HNC), especially nasopharyngeal carcinoma (NPC), is an aggressive cancer type with high incidence rate in Southern China [1]. The cancer incidence data collected in Guangxi and Guangdong show that nasopharyngeal cancer is the fourth most common cancer for males [2]. External beam radiation therapy is the primary therapy to this cancer. The 3-year local control rate for NPC after therapy is higher than 80% and the 3-year overall survival rate is up to 90% [3]. Noninvasive medical imaging is of great importance to determine the tumor volume for successful radiation treatment planning [3, 4].

Dynamic contrast-enhanced magnetic resonance imaging (DCE-MRI), a functional noninvasive imaging modality, plays a key role in the studies of cancer by providing information about physiological characteristics in tissues. Studies have concluded that DCE-MRI is useful in differentiating tumors from normal tissues in NPC [4]. Accurate segmentation of NPC tumors from DCE-MRI is important for the radiotherapy treatment planning and prognosis evaluation. However, the accuracy of tumor segmentation in DCE-MRI is affected by some imaging factors such as low spatial resolution, poor signal-to-noise ratio, partial volume effect, and the intensity changes during perfusion [5].

There have been many studies performed to automatically segment NPC tumors from medical images. Zhou et al. [6] performed NPC tumor segmentation in MR images by using Semi-Fuzzy C-means with the percent match (PM) values close to 0.87. Zhou et al. [7] performed NPC tumor segmentation in MRI by using the two-class support vector machine (SVM) method with PM values close to 0.79. Huang et al. [8] performed semisupervised NPC lesion extraction in MR images by using spectral clustering-based method with the positive predictive value up to 0.71. Huang et al. [9] performed NPC tumor segmentation by using Bayesian classifiers and SVM method with average specificity of 0.93.

The above-mentioned methods were all conventional machine learning techniques that require subjective feature extraction and selection. Deep learning (DL) technique, such as convolutional neural network (CNN), has recently emerged as a powerful tool in solving the challenges aforementioned, which detects low-level features such as shape and texture information autonomously from small patches of the input images and then combines these features into high-level features for the image processing tasks such as classification, segmentation, and detection without the subjective feature extraction and selection [10, 11]. Deep learning techniques perform even better in generalization with new datasets [12].

To the best of our knowledge, DL with CNN technique in tumor segmentation has recently attracted research interest [13, 14]. Wang et al. [15] performed NPC tumor segmentation in MR images by using deep convolutional neural networks; however, the average Jaccard similarity coefficient (JSC) value was less than 0.8. In the current study, we reported an automatic and accurate segmentation method based on the CNN architecture with dynamic contrast-enhanced MRI.

## 2. Materials and Methods

### 2.1. CE-MRI and Preprocessing

Twenty-nine newly diagnosed NPC patients from August 2010 to April 2013 were included from the First Affiliated Hospital, Sun Yat-Sen University. This study was approved by the local institutional review board of Sun Yat-Sen University. Written informed consent was obtained from each patient before the MRI scan. PVE could severely affect the images whenever the tumor size is less than 3 times the full width at half maximum (FWHM) of the reconstructed image resolution [16]. Thus, the patients with lymph nodes or lesions smaller than 1 cm were excluded in the current study to avoid possible partial volume effects (PVE), according to the advice from the radiologists. Imaging of DCE-MRI was performed in the primary tumor region including the retropharyngeal nodes with regional nodal metastasis, in with a 3.0-T MRI system (Magnetom Trio, Siemens) with the field of view of 22cm×22cm×6cm (AP×RL×FH), a flip angle of 15°, and scanning time of 6 minutes and 47 seconds, resulting in 65 dynamic images. The contrast agent gadolinium-diethylenetriamine pentaacetic acid (Gd-DTPA) (Omniscan; Nycomed, Oslo, Norway) was injected intravenously as a bolus into the blood at around the 8th dynamic acquisition using a power injector system (Spectris Solaris, MedRad, USA) and a 25 mL saline flush at a rate of 3.5 mL/sec was immediately followed. The dose of Gd-DTPA was 0.1 mmol per body weight in kg of the patient. The matrix of the 65 reconstructed dynamic image was 144×144×20×65.

The ground truth was manually contoured in ImageJ (National Institutes of Health, Bethesda, MD) with the consensus between two experienced radiologists (Dr. Yufeng Ye, 13 years' experience, and Dr. Dexiang Liu, 18 years' experience in Radiology) who were blind to this study. Since tumors were mostly enhanced at the 35^th^ scan of our DCE-MRI, this scan from each patient was used for training and testing our DL model, and we only selected the scanned images containing the tumor area. There were a total of 87 slices of CE-MRI acquired from each of the 29 patients. To fulfill the requirement of large number of data in training the DL model, we augmented the 87 MRI to more than 60,000 slices of images by using the following methods [17], namely, rotating each slice between -10 degrees and 10 degrees with an interval of 2 degrees to augment each slice to 11 slices, changing the image contrast with an embedded Matlab function, Imadjust, to adjust the image contrast automatically to produce 33 extra different slices from one single slice and adding Gaussian noise to the images with a power of 1×10^−8^ to produce 2 different additional slices from each slice. Totally we augmented the images by 11×33×2=726 times for each patient's CE-MRI set to give a total of 63126 (87x726) slices. These augmented images were then normalized by performing Z-score translation [18], in which the image intensity value in each voxel was normalized by the mean intensity of this image.

### 2.2. CNN Network

**Figure 1:**
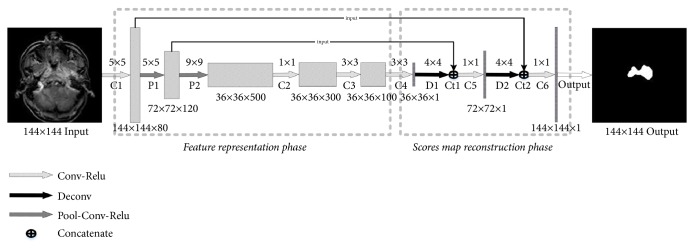
The schematic diagram of the proposed convolutional neural network (CNN) structure. The proposed CNN network includes two phases of feature representation and scores map reconstruction. The feature representation phase consists of 2 Pool-Conv-ReLu blocks and 3 Con-ReLu blocks, while the scores map reconstruction phase consists of 2 deconv-concat-conv blocks. The output of each layer is a three-dimensional matrix with size of h×w×d, where h and w are the length and width of the scores map, respectively, and d is the feature dimension. a×a indicates the matrix size of the convolution kernels. Conv: convolution, Relu: rectified linear units, Pool: pooling, Deconv: deconvolution.

The CNN network included two phases of feature representation and scores map reconstruction. In the feature representation phase, the network consisted of 2 Pool-Conv-ReLu blocks (P1-P2) and 4 Conv-ReLu blocks (C1-C4) (see Figure 1). A Pool-Conv-ReLu block included one pooling layer (Pool), one convolution layer (Conv), and one rectified linear units (ReLu) layer, while a Conv-ReLu block consisted of one convolution layer and one ReLu layer. The convolution layer detected local features from the input images and the ReLu layer accelerated the convergence. The pooling layer was designed for reducing the dimension of feature maps and network parameters. The input images with a matrix size of 144×144 were transformed into the feature maps of matrix size of 36×36 in the feature representation phase.

In the scores map reconstruction phase (D1-D2, Ct1-Ct2, C5-C6), the images were reconstructed from the 36×36 feature maps. Two deconvolution layers (D1-D2) were applied to reconstruct an output image with a matrix size of 144×144. Since some image details could be missing in this reconstruction from the 36×36 feature maps, the fine features obtained from the previous feature representation phase were combined with the scores map to allow the integration of local and global multilevel contextual information. A concatenate layer was then used for the information connection. Then a convolution layer was applied for information fusion and the final reconstruction. The detailed parameters of the CNN network are shown in Table 1.

### 2.3. Model Training and Model-Based Segmentation

A stepwise training scheme was used to train the DL CNN network. Firstly, we trained the network in the feature representation phase and a 36×36 scores' map was obtained. Based on this 36×36' scores map, we next trained the deconvolution layer, the concatenate layer, and the convolution layer, resulting in a 72×72 scores' map. Finally, based on the network parameters and output feature maps acquired in the second step, we reconstructed the images with matrix size of 144×144 scores' map.

In the training process, the weights were optimized in each iteration. The weight of a Gaussian distribution with mean of 0 and standard deviation of 1 was used in the convolution kernel at the initialization step. The training parameters were as follows: basic learning rate: 1×10^−7^, step size: 1x10^5^, gamma: 0.1, momentum: 0.9, weight decay: 5x10-4. It took 52 hours for a complete training procedure with a NVIDIA GeForce GTX 980 GPU equipped on an Intel Core i7 3.5 GHz computer.

We used the trained model in the segmentation tasks of NPC tumor lesions in the testing dataset. The testing images were input into the trained model. A score map representing the tumor region of the NPC tumor was acquired for each input image.

### 2.4. Tumor Segmentation

We used the testing dataset to make forward propagation and evaluated the segmentation performance based on the trained model. Parameters of recall, precision, and dice similarity coefficient (DSC) were given by

(1) recall=TPTP+FP

(2) precision=TPTP+FN

(3) DSC=2∗11/recall+1/precision

where true positive (TP) denotes the correctly identified tumor area and false positive (FP) denotes the tumor area, but the area is normal tissue in ground truth and false negative (FN) denotes normal tissue but the pixel isolated is tumor area in ground truth. And those are the results for each patient.

For the comparisons with other published results, values of corresponding ratio (CR), percent match (PM) [7], and Jaccard similarity coefficient (JSC) [15] were also calculated as

(4) CR=TP−0.5×FPTP+FN

(5) PM=TPTP+FN

(6) JSC=TPTP+FP+FN

The model validation technique of leave-one-out cross-validation (LOOCV) was used such that, in one repetition, the images of 28 patients were used as the training dataset (which were then augmented to >60000 images), and the images of the remaining patient were used as the testing dataset. After each patient's images in these 87 images were tested, the mean and variance of DSC, recall, CR, PM, and JSC were calculated to evaluate the segmentation performance of our method.

## 3. Results

Table 2 tabulates the tumor volumes as segmented by the radiologist (the golden standard) and by the proposed automatic segmentation method together with DSC, CR, PM, recall, and JSC. These values were calculated for each patient, not for each lesion.  Table 3 shows the comparison of segmentation performance in terms of DSC, CR, and PM between our current results of DL CNN network and those of published results using other models. The mean DSC with our method for 29 patients was 0.89±0.05, and the range was 0.80-0.95. The mean PM with our method for 29 patients was 0.90±0.04 with a range of 0.71-0.92, which was higher compared to the mean PM of the value less than 0.9 in other studies. The mean CR was 0.84±0.06 and the range was 0.83-0.96, while the mean CR was 0.72 in similar studies using other algorithms [7, 15, 19].

**Figure 2:**
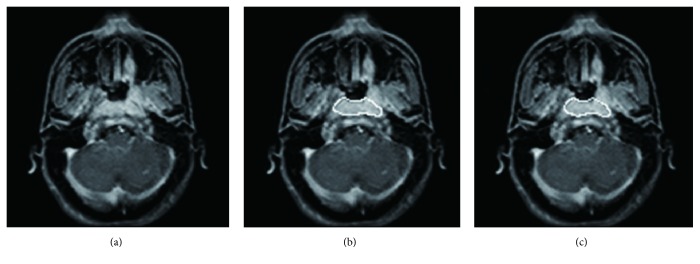
NPC tumor segmentation with high accuracy using the current deep learning method with convolutional neural network. (a) The original image. (b) Ground truth (white line). (c) Segmentation from our deep learning method result (white line) with the dice similarity coefficient = 0.941, corresponding ratio = 0.915, and percent match = 0.950.

Figure 2 shows the segmentation with high accuracy, in which the DSC, CR, and PM were 0.941, 0.915, and 0.950, respectively, showing good accordance between segmentation results using our current DL CNN network and ground truth.

**Figure 3:**
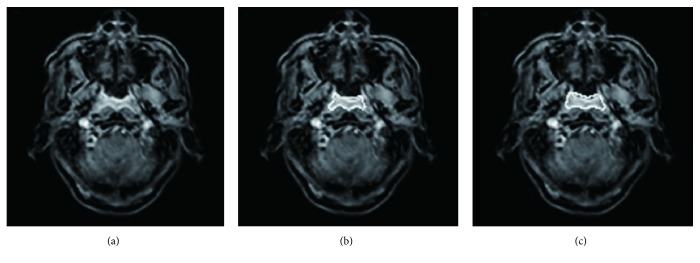
NPC tumor segmentation with less accuracy using the current deep learning method with convolutional neural network. (a) The original image. (b) Ground truth (white line). (c) Segmentation from our deep learning method result (white line) with the dice similarity coefficient = 0.797, corresponding ratio = 0.731, and percent match = 0.937.

Figure 3 shows a less accurate segmentation result as obtained by our current DL CNN model with values of DSC, CR, and PM being 0.797, 0.731, and 0.937, respectively, showing slight difference between segmentation results using our current DL CNN network and ground truth.

## 4. Discussion

Based on the CNN technique, we achieved a supervised segmentation method for NPC tumors in CE-MRI with high accuracy of mean DSC being 0.89. The performance was also robust with a low standard deviation of 0.05 for DSC among the results of different images. For comparison with the other studies, we calculated CR and PM. The mean values of CR and PM achieved with our method were 0.84 and 0.90, respectively. Compared with similar studies in literature, results of CR and PM in our study are more superior, indicating better accuracy in tumor segmentation with our current CNN technique than with other models with highest mean CR and PM of 0.72 and 0.90, respectively [7, 15, 19]. This may indicate that our method has indeed improved the automatic segmentation accuracy.

Firstly, the improvement may lie in the application of CNN to extract the image features automatically and objectively. In our model, the low-level features were combined into high-level features with semantic information through convolutions (Figure 1). By iterations through the back propagation algorithm, we highlighted the characteristics associated with the targeted area and gradually suppressed irrelevant features [12]. In this way, our model can extract the most useful features and achieve better segmentation results.

**Figure 4:**
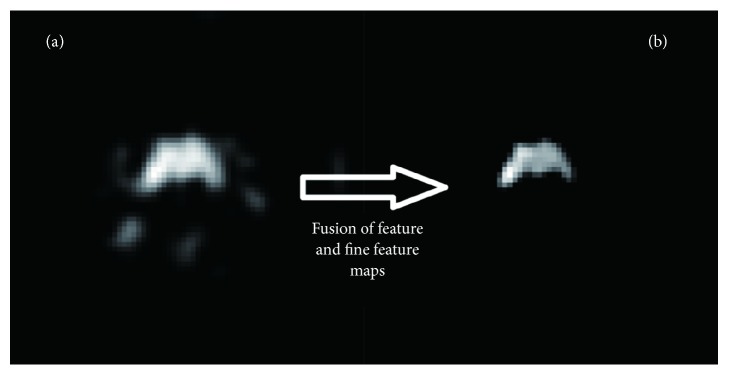
A feature map fusion. (a) Scores map acquired in the reconstruction phase and (b) scores map reconstructed from the fused feature maps, indicating that score map reconstruction using fused maps is better than that during reconstruction phase.

Secondly, in our designed network architecture, we fused the different feature maps at feature representation phase and scores map reconstruction phase for the final reconstruction. As shown in Figure 4(a), which was acquired in the reconstruction phase, the tumor location and shape are roughly visible; however, they are unclear. Through the fusion of this feature map and the fine-feature map acquired in feature representation phase, we may fix the problem of information loss in the reconstruction process. As shown in Figure 4(b), we finally had better segmentation through the reconstruction from the fused feature maps.

There is space to further improve the accuracy and effectiveness of our current model. As shown in Table 3, our method resulted in less accurate DSC results of 0.80 in some cases. For further improvement, we may include T2 weighted images, since T2 weighted images are widely used in the manual contouring of tumor regions. Therefore we would expect to have better performance with both the DCE-MRI with T2 weighted images. We applied the Z-score translation in preprocessing to normalize the DCE-MRI [18]. However, some information could be lost during this normalization. Therefore, we may investigate an appropriate method of normalization to avoid the loss of intrinsic image features. Importantly, we may improve our network architecture, such as the depth of our network, for the direct training of the 3D images and the incorporation of time domain information from the dynamic scans. In future studies, it is expected to further improve our method and the segmentation results with these ideas.

## 5. Conclusion

A robust segmentation method for NPC tumor based on deep learning convolutional neural network and CE-MRI has been established. The tumors can be segmented successfully in seconds with high accuracy. This automatic segmentation method may be time-effective in tumor contouring for routine radiotherapy treatment planning. Future studies may aim to improve the segmentation accuracy and efficiency with more training data and optimized network structure, thus helping clinicians improve the segmentation results in the clinical practice of NPC.

## Figures and Tables

**Table 1:** Detailed parameters of the CNN network.

**Block**	**Layer**	**Kernel size**	**Stride**	**Pad**	**Output size**
**Input Data**	-	-	-	-	144*∗*144*∗*1
**Conv-Relu 1**	Conv1	5	1	2	144*∗*144*∗*80
	Relu1	-	-	-	144*∗*144*∗*80
**Pool-Conv-Relu 1**	Pool1	2	2	-	72*∗*72*∗*80
	Conv2	5	1	2	72*∗*72*∗*120
	Relu2	-	-	-	72*∗*72*∗*120
**Pool-Conv-Relu 2**	Pool2	2	2	-	36*∗*36*∗*120
	Conv3	9	1	4	36*∗*36*∗*500
	Relu3	-	-	-	36*∗*36*∗*500
**Conv-Relu 2**	Conv4	1	1	0	36*∗*36*∗*300
	Relu4	-	-	-	36*∗*36*∗*300
**Conv-Relu 3**	Conv5	3	1	1	36*∗*36*∗*100
	Relu5	-	-	-	36*∗*36*∗*100
**Conv-Relu 4**	Conv6	3	1	1	36*∗*36*∗*1
	Relu6	-	-	-	36*∗*36*∗*1
**Deconv1**	Deconv1	4	2	1	72*∗*72*∗*1
**Concatenate1**	Concat1	-	-	-	72*∗*72*∗*121
**Conv-Relu 5**	Conv7	1	1	0	72*∗*72*∗*1
	Relu7	-	-	-	72*∗*72*∗*1
**Deconv2**	Deconv2	4	2	1	144*∗*144*∗*1
**Concatenate2**	Concat2	-	-	-	144*∗*144*∗*81
**Conv6**	Conv8	1	1	0	144*∗*144*∗*1
**Output**	Flatten1	-	-	-	144*∗*144*∗*1

**Table 2:** The segmentation performance of all cases.

Patients number	Volume of current DL method (cm^3^)	Volume obtained by the two readers (cm^3^)	Percent match	Corresponding ratio	Dice similarity coefficient	Recall	Jaccard similarity coefficient
1	7.8	8.0	0.84	0.74	0.83	0.81	0.70
2	5.2	6.2	0.90	0.76	0.82	0.75	0.70
3	5.1	6.4	0.89	0.72	0.80	0.72	0.66
4	18.5	18.4	0.88	0.83	0.89	0.89	0.79
5	7.0	6.5	0.86	0.82	0.89	0.92	0.80
6	11.9	10.6	0.85	0.82	0.89	0.94	0.81
7	11.9	11.9	0.83	0.74	0.83	0.82	0.71
8	11.0	10.2	0.85	0.82	0.89	0.92	0.80
9	5.9	6.7	0.90	0.78	0.84	0.79	0.73
10	14.8	17.1	0.86	0.71	0.80	0.75	0.66
11	4.4	4.7	0.96	0.91	0.93	0.91	0.87
12	17.3	17.9	0.94	0.89	0.92	0.90	0.85
13	4.5	4.3	0.88	0.84	0.90	0.92	0.82
14	46.7	45.0	0.93	0.91	0.95	0.97	0.90
15	5.8	5.7	0.88	0.82	0.88	0.89	0.79
16	8.8	9.2	0.90	0.83	0.88	0.87	0.79
17	5.7	6.8	0.92	0.79	0.84	0.78	0.73
18	10.7	11.2	0.94	0.89	0.92	0.90	0.85
19	10.6	10.9	0.95	0.91	0.94	0.93	0.88
20	8.6	8.9	0.93	0.88	0.91	0.90	0.84
21	13.3	13.9	0.93	0.87	0.91	0.89	0.83
22	13.7	13.6	0.95	0.92	0.95	0.95	0.90
23	6.3	6.5	0.90	0.83	0.88	0.87	0.79
24	16.2	16.5	0.95	0.92	0.94	0.94	0.89
25	18.2	17.7	0.94	0.92	0.95	0.96	0.91
26	11.4	11.1	0.91	0.87	0.92	0.93	0.85
27	10.8	11.0	0.88	0.80	0.87	0.86	0.76
28	17.1	17.9	0.95	0.90	0.93	0.91	0.86
29	12.0	12.2	0.94	0.90	0.93	0.92	0.87
Mean±Std	-	-	0.90±0.04	0.84±0.06	0.89±0.05	0.88 ±0.07	0.81 ±0.07

**Table 3:** Comparison of segmentation performance among our convolutional neural network model and other models. N.A.: not available.

		Dice similarity coefficient		Corresponding ratio		Percent match	
Study	Algorithm	Mean ± SD	Range	Mean ± SD	Range	Mean ± SD	Range
Current study	Convolutional neural network	0.89±0.05	0.80-0.95	0.84±0.06	0.71-0.92	0.90±0.04	0.83-0.96
Zhou at al. [7]	Support vector machine	N.A	N.A	0.72±0.06	0.58~0.85	0.79±0.07	0.65-0.91
Huang et al. [14]	Hidden Markov random field	N.A	N.A	0.72	0.44-0.91	0.85	0.64-1.00
Wang et al. [15]	Deep Convolutional Neural Networks	N.A	-0.80	N.A	N.A	N.A	-0.90

## Data Availability

The authors do not have permission to share data.
